# Bioinformatic identification of hub genes and related transcription factors in low shear stress treated endothelial cells

**DOI:** 10.1186/s12920-021-00971-6

**Published:** 2021-05-03

**Authors:** Yang Yang, Xiangshan Xu

**Affiliations:** Cardiology Department Fourth Affiliated Hospital of China Medical University, Fourth Chongshan East Road, Huanggu District, Shenyang, 110032 Liaoning China

**Keywords:** Low shear stress, Differentially expressed genes, Bioinformatics, Transcriptional factors

## Abstract

**Background:**

Recent evidences indicated that shear stress is critical in orchestrating gene expression in cardiovascular disease. It is necessary to identify the mechanism of shear stress influencing gene expression in physiology and pathophysiology conditions. This paper aimed to identify candidate hub genes and its transcription factors with bioinformatics.

**Methods:**

We analyzed microarray expression profile of GSE16706 to identify differentially expressed genes (DEGs) in low shear stress (1 dyne/cm^2^) treated human umbilical vein endothelial cells (HUVECs) compared with static condition for 24 h.

**Results:**

652 DEGs, including 333 up-regulated and 319 down-regulated DEGs, were screen out. Functional enrichment analysis indicated enrichment items mainly included cytokine-cytokine receptor interaction and cell cycle. Five hub genes (*CDC20*, *CCNA2*, *KIF11*, *KIF2C* and *PLK1*) and one significant module (score = 17.39) were identified through protein–protein interaction (PPI) analysis. Key transcriptional factor *FOXC1* displayed close interaction with all the hub genes via gene-transcriptional factor network. Single-gene GSEA analysis indicated that *CDC20* was linked to the G2M_CHECKPOINT pathway and cell cycle pathway.

**Conclusions:**

By using integrated bioinformatic analysis, a new transcriptional factor and hub-genes network related to HUVECs treated with low shear stress were identified. The new regulation mechanism we discovered may be a promising potential therapeutic target for cardiovascular disease.

## Background

Atherosclerosis is the main causes of cerebrovascualr and cardiovascular disease around the world [1]. The risk factor of atherosclerosis contains many conventional risk factors, including diabetes, smoking, hypertension, obesity, hypercholesterolemia and family history [2]. In addition, mounting evidence indicated that atherosclerosis was prone to occur at the curve or bench region of the vascular, where was characterized by disturbed blood flow with low wall shear stress [3]. Thin-cap fibroatheroma, which is prone to locate in the proximal of coronary artery characterized by low shear stress, was associated with plaque rupture in coronary artery disease [4]. This phenomenon suggested that low shear stress played a pivotal role in the initiation and progression of atherosclerosis [5]. This link between atherosclerosis progression and low shear stress is very well established and has been known for many decades [6]. Several mechanosensitive biomarkers have been considered to be associated with low shear stress in endothelial cells [7]. However, the mechanism of shear stress regulating gene expression in endothelial cells remains not be fully understood.

Transcription factor (TFs) is a protein that can bind to specific DNA sequences to regulate multiple biological processes including cell differentiation, cell cycle regulation, stress responses, cell proliferation and apoptosis [8]. It can form complex with other proteins or TFs alone, modulating the expression of genes by activating or repressing the recruitment of RNA polymerase [9]. Thanks to the rapid development of array technology and next generation sequencing technology, it makes bioinformatic analysis method an important way to identify new biomarkers in various disease, including cancer, metabolic disease and cardiovascular disease. For instance, Hu [10] identified TFs and its binding sites via characteristic domain analysis method in genome wide. Meanwhile, researches could screen out regulatory networks participating in many biological and molecular processes in co-expression studies.

In the present study, we firstly selected the differentially expressed genes (DEGs) in human umbilical vein endothelial cells (HUVECs) exposed to low shear stress treatment comparing with static treatment. Then, we analyzed the biological function of DEGs via Gene ontology (GO) [11] and Kyoto Encyclopedia of Genes and Genomes (KEGG) [12] pathway analyses. The protein–protein interaction (PPI) network was constructed through the search tool for the retrieval of interacting genes (STRING) website [13] to find hub genes and the results were displayed with Cytoscape software [14]. Last, we established the gene-TFs regulation network to identify the TFs which participated in the regulation of ECs under low shear stress treatment. The relationship between *CDC20* and *FOXC1* was further validated by RT-PCR method. Based on these results, it provided new insight of TFs-gene regulation pathway in atherosclerosis and potential therapeutic target of cardiovascular diseases.

## Methods

### ECs cell culture, transfection and low shear stress treatment

HUVECs were supplied by China Infrastructure of Cell Line Resource (Beijing). The culture condition contains Dulbecco modified Eagle medium (DMEM), 37 °C and 5% CO2 atmospheres. When the density reached 70–80%, ECs were transiently transfected with *FOXC1* siRNA or scrambled control supplied by Sangon Biotech (Biotech (Shanghai) Co., Ltd. Shanghai, China) at the concentration of 100 nM to down-regulate the expression of *FOXC1*. The sequence of *FOXC1* siRNA or the scrambled control was validated by the company. After the transfection was successfully performed, ECs were continued cultured in the shear stress treatment simulating system (Shanghai Naturethink life science & Technology CO, Ltd) treated with low shear stress (5 dyne/cm^2^) lasting 24 h. The control group was cultured under static condition for 24 h, too.

### Data acquisition and preprocessing

First of all, we searched datasets focusing on the gene expression profile related to low shear stress treatment via GEO database. According to the selection criteria, GSE16706 emerged from the GEO database which included low shear stress and static treatment in HUVECs. Dataset GSE16706 was annotated by GPL6480. GSE 16706 consisted of 3 low shear stress treatment samples, 3 high shear stress treatment group, 3 reverse treatment samples and 3 static control treatment samples. Considering low shear stress is a prone atherosclerotic factor, in this paper we selected low shear stress treatment samples (GSM418608, GSM418612 and GSM418616) and static control treatment samples (GSM418611, GSM418615 and GSM 418619) for further study. The experimental protocol in GSE16706 dataset: HUVECs were mounted in a parallel plate flow chamber. After that the HUVECs were exposed to 1 dyne/cm^2^ shear stress treatment or static culture for 24 h. Ending exposure to shear stress treatment, total RNA was prepared via TRI-Zol method (Invitrogen) according to manufacturers’ instructions. The gene expression was detected using corresponding annotation platform according to the manufactures’ instruction. Three independent experiments were performed in each group.

R/Bioconductor package GEOquery [15] was used to download the gene expression data from GEO website. The downloaded data from GEO datasets consisted of probe ID and expression matrix. Then probe ID was converted to corresponding gene symbols according to the annotation of GPL6480. When multi-probes were matched to the same gene, we selected the maximum value of theses probes as the expression level for the following analysis. PreprocessCore package of R was used to perform quantile normalization and background correction.

### Identification of DEGs

Limma package [16] was applied to identify DEGs between low shear stress treatment group and static control group. Briefly, DEGs were selected according to the following criteria: adjusted P-value < 0.05 and absolute log2 fold change (FC) value > 2. Volcano plots comparing log10 (statistical relevance) to log2 FC were generated using R software (version3.6.3, AT&T Bell Laboratories, New York, NY, USA). We visualized DEGs via heatmap generated by the pheatmap package in R software.

### GO and KEGG pathway analyses

To better research and demonstrate the biological function of DEGs, we routinely performed GO analysis, which included biological process (BP), cellular component (CC) and molecular function (MF). In addition, signal pathway analysis of DEGs was also carried out according to the latest KEGG database [12]. The clusterProfiler package in R software was utilized in performing GO and KEGG analysis. When *P* value was below 0.05, we considered the results had statistical significance.

### Establishment and analysis of PPI network

STRING (https://string-db.org/) is a widely used website to study the protein–protein interaction information and is also broadly adopted to identify hub genes according to the connectivity and node parameter. In this paper, we also established the PPI network according to the latest STRING database [13]. We selected node pairs with a combined score ≥ 0.4 in the PPI network for next step analysis. Following that, the protein–protein interaction results were displayed by Cytoscape software. The topological parameters of each gene in the PPI network were analyzed to identify hub genes. Next, Molecular Complex Detection (MCODE) [17] was utilized to find out the most important modules among PPI network. The cutoff value was set as: node score cutoff = 0.2, K-Core = 2, and degree cutoff = 2.

### Establishment of TFs-hub genes network

Network Analyst [18] (http://www.networkanalyst.ca/faces/home.xhtml) was used in this paper to analysis the TFs-gene interactions. By this analysis we could further investigate the effect of TFs on the target hub genes. In the present bioinformatic analysis, we predicted the TFs of identified hub genes. Meanwhile, the TFs-genes regulatory results were established and displayed by Cytoscape software.

### Gene set enrichment analysis (GSEA)

We performed GSEA analysis in order to investigate the potential function of hub genes in the TFs-genes network [19]. Briefly, according to the average expression level of the low shear stress treated group, the Spearman correlation coefficient between *CDC20* and other genes in low shear stress treatment group was calculated and sorted by Spearman correlation coefficient. Then the clusterProfiler package [20] was adopted to complete the single gene GSEA analysis. *P* value < 0.05 means the results have significant difference.

### Validation of target gene by qRT-PCR assay

The mRNA expression profile of target genes was evaluated through RT-PCR method. Briefly, total RNAs were extracted from HUVECs with FastPure Cell/Tissue Total RNA Isolation Kit (Vazyme, Nanjing, China) following the manufacturer’s instruction. Then total RNA was reverse transcribed to cDNA with HiScript II 1st Strand cDNA Synthesis Kit (Vazyme, Nanjing, China). The mRNA expression level of *FOXC1* and *CDC20* was detected with ChamQ Universal SYBR qPCR Master Mix (Vazyme, Nanjing, China). *GAPDH* was used as house-keeping gene. 2^−ΔΔCT^ method was performed to compare the gene expression level between low shear stress treated group and control group. The protocol of PCR: 5 min at 95 °C, 40 cycles of 95 °C for 10 s and 60 °C for 30 s. The sequences of primer pairs are as follows:

- *CDC20*: F: 5’-GGCAGTCCAATGTCC-3’; R: 5’-GGAGACCAGAGGATGGAGCAC-3’
- *FOXC1*: F: 5’- TTCTTGCGTTCAGAGACTCG-3’; R: 5’- TCTTACAGGTGAGAGGCAAGG-3’
- *GAPDH*: F: 5’-CATACCAGGAAATGAGCTTG-3’, R: 5’-ATGACATCAAGAAGGTGGTG-3’

### Measurement of apoptosis in ECs by flow cytometry assay

The apoptosis of HUVECs transfected with siFOXC1 or scrambled control treated with or without low shear stress treatment was evaluated using Annexin V-PE/7-AAD Apoptosis Detection Kit (Vazyme, Nanjing, China) according to the manufacture’s instruction. The results were detected by FACS (BD Celesta). Flowjo software 7.6 was used to analyze the data.

### Statistical analysis

All the statistical analyses were performed in R software and *P* value < 0.05 was considered statistically significant. The differences between two groups was analyzed using non-parametric test or t test according to the data distribution characteristics.

## Results

### Data acquisition and DEGs identification

**Fig. 1:**
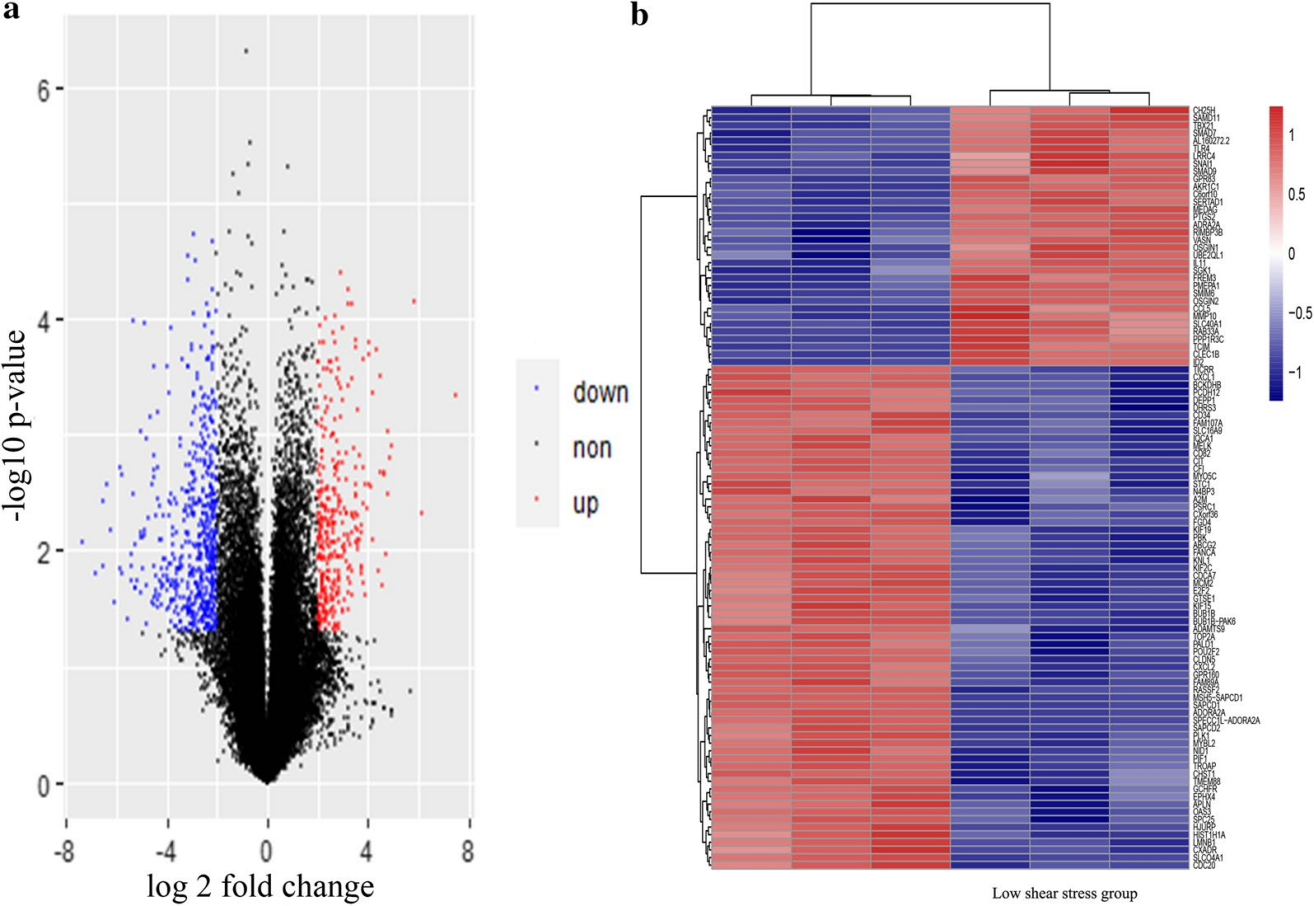
The volcano plots and heat map showing expression profiles of GSE16706. **a** The volcano map of GSE16706. Red dot indicates genes with high levels of expression, blue indicates genes with low levels of expression, and gray indicates genes with no differential expression based on the criteria of *P* value < 0.05 and |log2 FC|> 2.0. **b** Heatmap of top 100 DEGs in GSE16706. Gene expression levels were indicated by colors as shown by the row, red represents high expression level and blue represents low expression level

The microarray expression matrices of GSM418608, GSM418612, GSM418616, GSM418611, GSM418615 and GSM 418,619 were successfully downloaded. By calculating the log2 FC and adjusted *p* value, we identified 652 DEGs including 333 up-expressed and 319 down-expressed according to the selection criteria. The results of microarray expression matrix were displayed by volcano graph and each plot in the volcano graph represented a gene in the expression matrix, and red represents the log2 fold change is above 2 and p-value is below 0.05, blue represents the log2 fold change of gene is below -2 and p-value is below 0.05 while gray suggested genes has no statistical significant differential expression or absolute fold change is below 2 (Fig. 1a). DEGs expression with the top 100 significant genes was displayed with heatmap in Fig. 1b.

### Functional and pathway enrichment analysis of DEGs

**Fig. 2:**
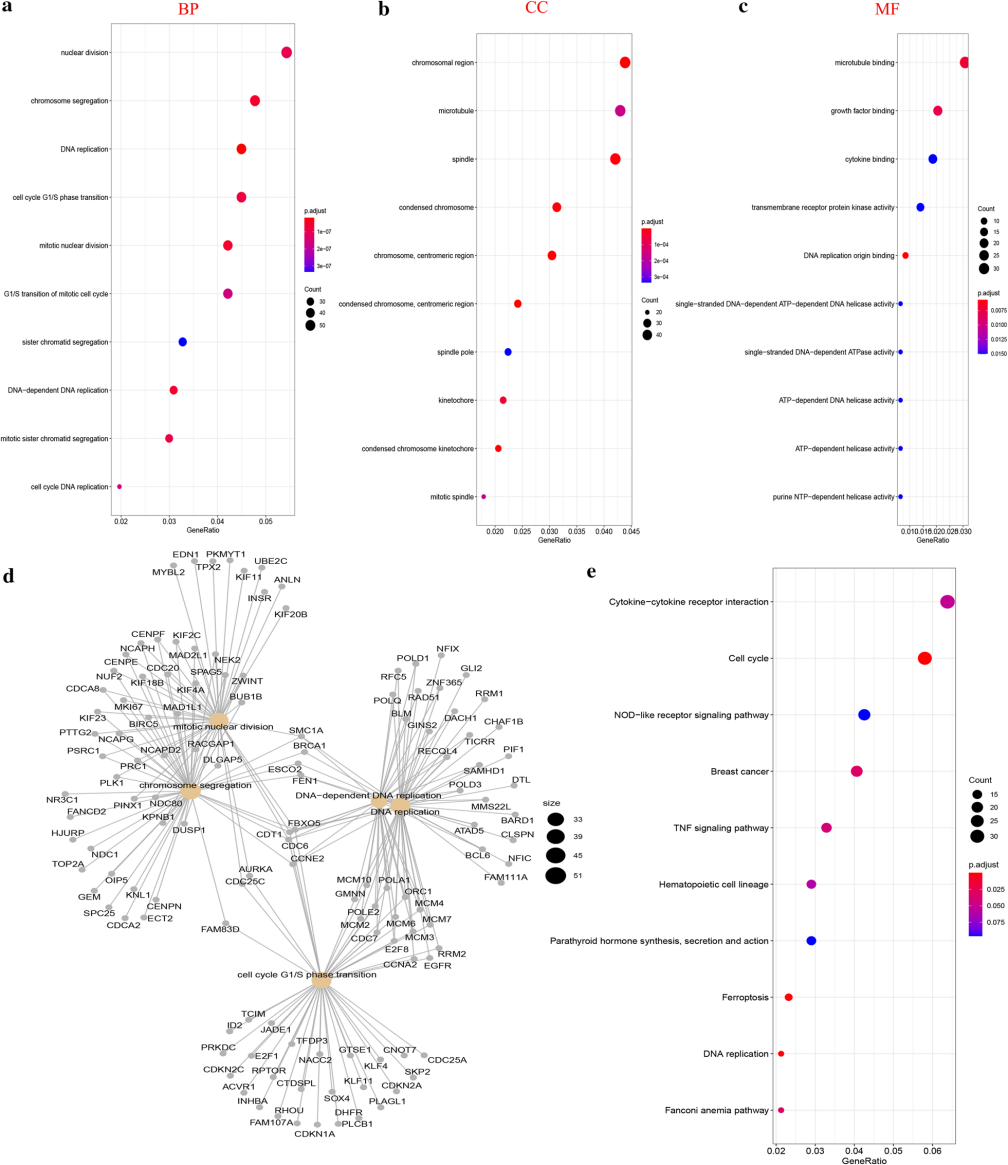
GO and KEGG analysis of DEGs. **a** GO-BP analysis of DEGs. **b** GO-CC analysis of DEGs. **c** GO-MF analysis of DEGs. **d** Network of the enriched terms and pathways. Nodes represent enriched terms or pathways with node size indicating the number of DEGs involved in. Nodes sharing the same cluster are typically close to each other. **e** KEGG pathway analysis of DEGs

Terms or pathways of GO and KEGG analysis with adjusted *P* value < 0.05 were selected. The most significant 10 enriched GO terms were displayed in Fig. 2. The results of Fig. 2a–c indicated that GO BP enriched terms of DEGs were mainly enriched in nuclear division, chromosome segregation, DNA replication, cell cycle G1/S phase transition and etc. GO CC analysis was significantly enriched in chromosomal region, microtubule, spindle and condensed chromosome. The main enriched MF terms included microtubule binding, growth factor binding, cytokine binding and transmembrane receptor protein kinase activity. Furthermore, we analyzed the network of enriched GO terms and related genes, As shown in Fig. 2d, the results indicated that there existed complicated connection between DEGs and GO annotation terms, which meant that many genes may play pivotal and multiple functions in cell cycle and cell division.

As listed in Fig. 2e, these KEGG pathways mainly included Cytokine-cytokine receptor interaction, Cell cycle, NOD-like receptor signaling pathway, TNF signaling pathway and DNA replication. Among the KEGG pathways identified from the KEGG analysis, cell cycle pathway possessed the most number of gene counts and the highest statistical significance.

### PPI network and identification of hub genes

**Fig. 3:**
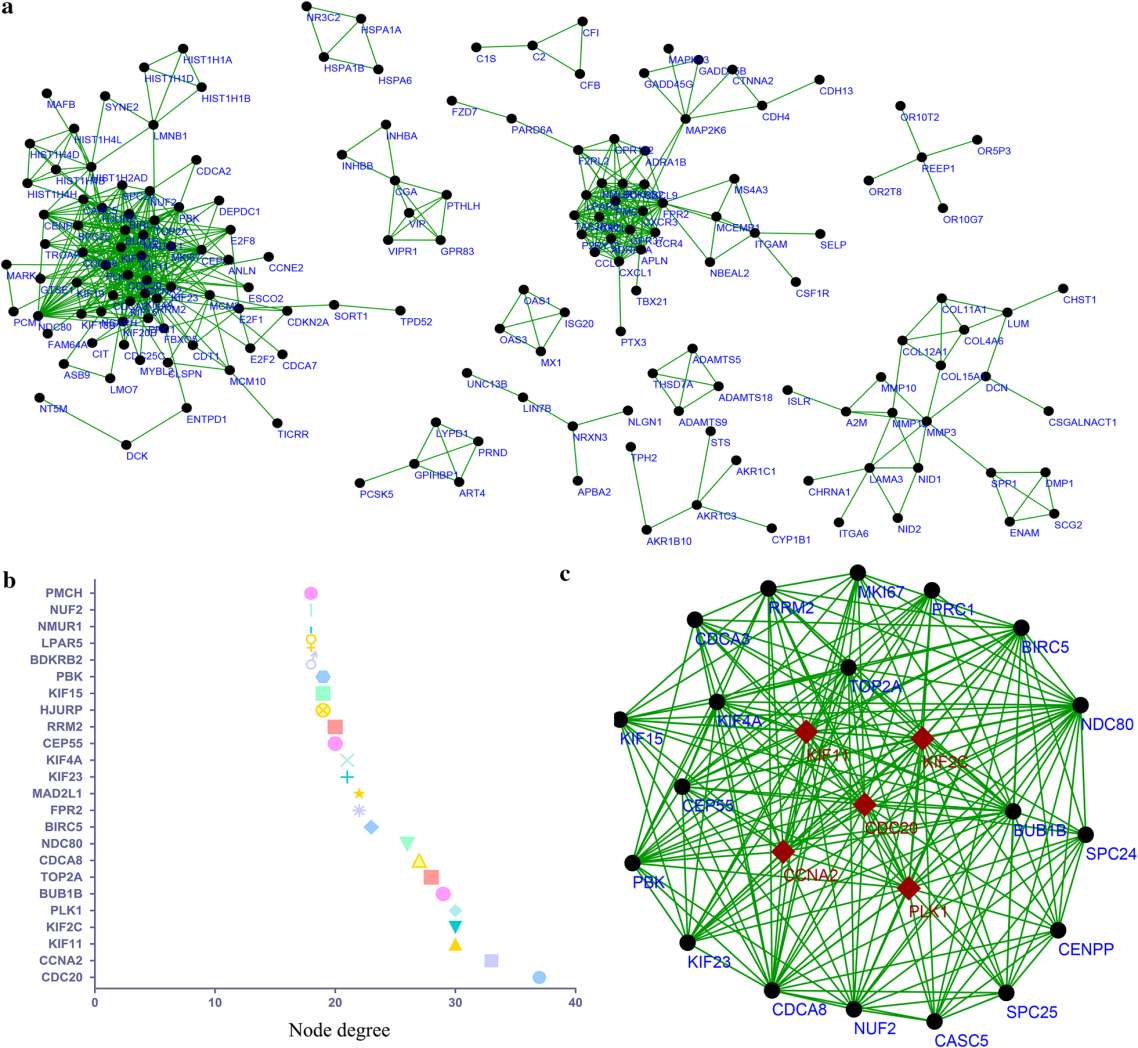
The protein–protein interaction (PPI) networks of DEGs. **a** The PPI network of total 652 DEGs. **b** The 24 genes with highest node degree. **c** The most significant module obtained from PPI network of DEGs (MCODE score = 17.39). The red color squares represents the hub genes of the DEGs

After PPI network was constructed by STRING website, the results were further visualized by Cytoscape software in Fig. 3a. This PPI network was made up of 228 DEGs and this network was consisted of 660 interaction pairs among these 228 nodes. The node degree was calculated to evaluate the importance of DEGs in the PPI network and the 24 highest node-degree genes constitute one sub-network (Fig. 3b). By comparing the network topology parameters, cell division cycle 20 (*CDC20*; degree = 37, betweenness centrality = 0.149, closeness centrality = 0.607), cyclin A2 (*CCNA2*; degree = 33, betweenness centrality = 0.176, closeness centrality = 0.602), kinesin family member 11 (*KIF11*; degree = 30, betweenness centrality = 0.051, closeness centrality = 0.573), kinesin family member 2C (*KIF2C*; degree = 30, betweenness centrality = 0.038, closeness centrality = 0.563), and polo like kinase 1 (*PLK1*; degree = 30, betweenness centrality = 0.147, closeness centrality = 0.582) were identified as hub geness (Fig. 3c).

### Functional analysis of module analysis

**Fig. 4:**
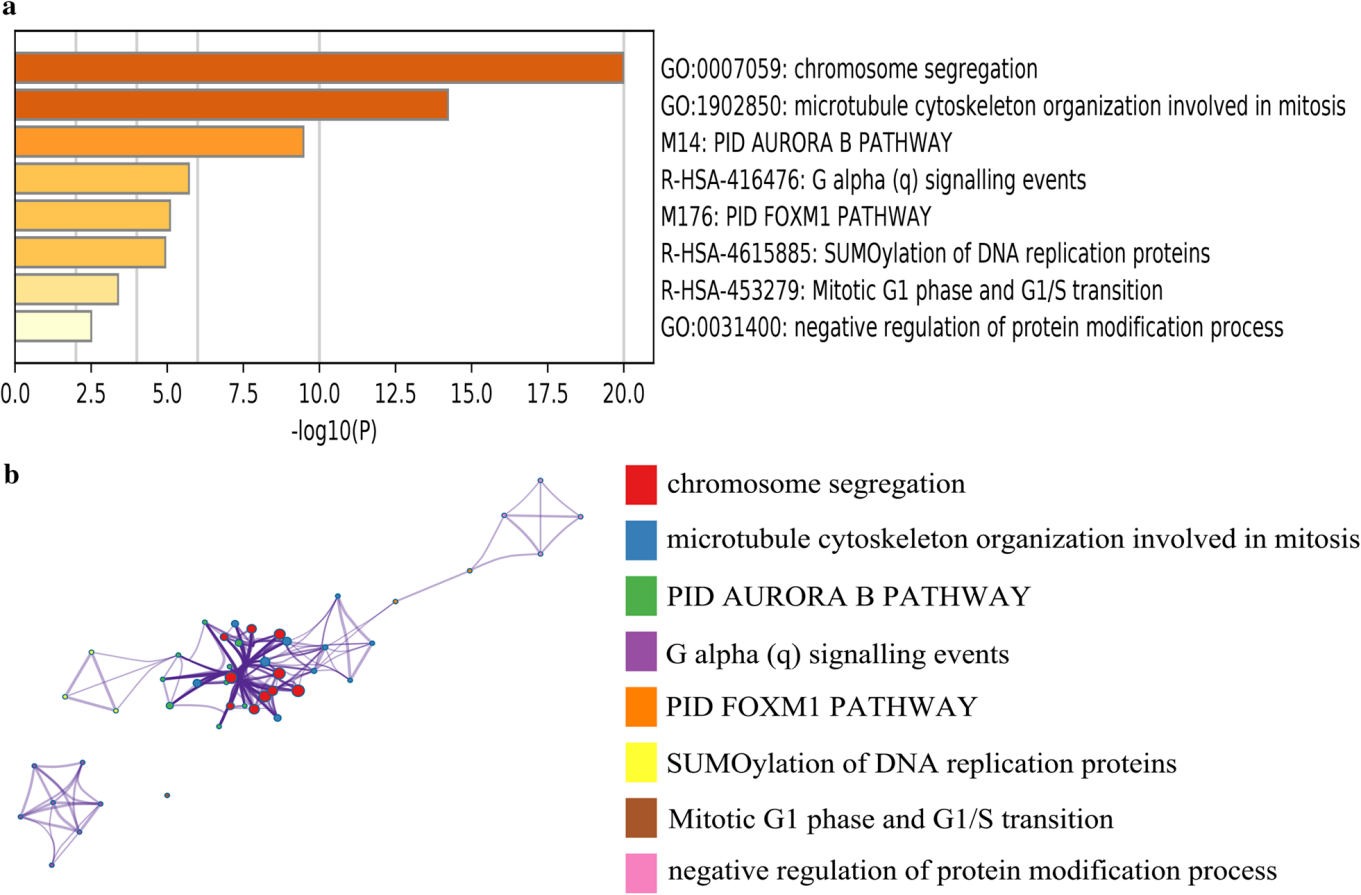
Functional and pathway enrichment analysis of module derived from PPI network. **a** GO terms and KEGG pathway were presented, and each band represents one enriched term or pathway colored according to the − log 10 P value. **b** Network of the enriched terms and pathways. Nodes represent enriched terms or pathways with node size indicating the number of DEGs involved in. Nodes sharing the same cluster are typically close to each other, and the thicker the edge displayed, the higher the similarity is

According to the MCODE analysis results, we screened out 17 significant models among the PPI network. Furthermore, we chose the most important module (MCODE score = 17.39) which contained the five hub genes as described before. This module included 24 nodes and 200 edges, and the entire hub DEGs were in the module. Next, we used the 24 genes in the module to perform GO and KEGG analysis by Metascape online website. The results suggested that these genes were mainly enriched in chromosome segregation and microtubule cytoskeleton organization involved in mitosis. In addition, AURORA B PATHWAY and G alpha signaling events pathway were enriched of these genes (Fig. 4a). Furthermore, network graph displayed that these identified enriched terms displayed closely connection with each other and these terms also clustered into intact networks (Fig. 4b).

### Transcriptional factor regulatory network analysis of hub genes

**Fig. 5:**
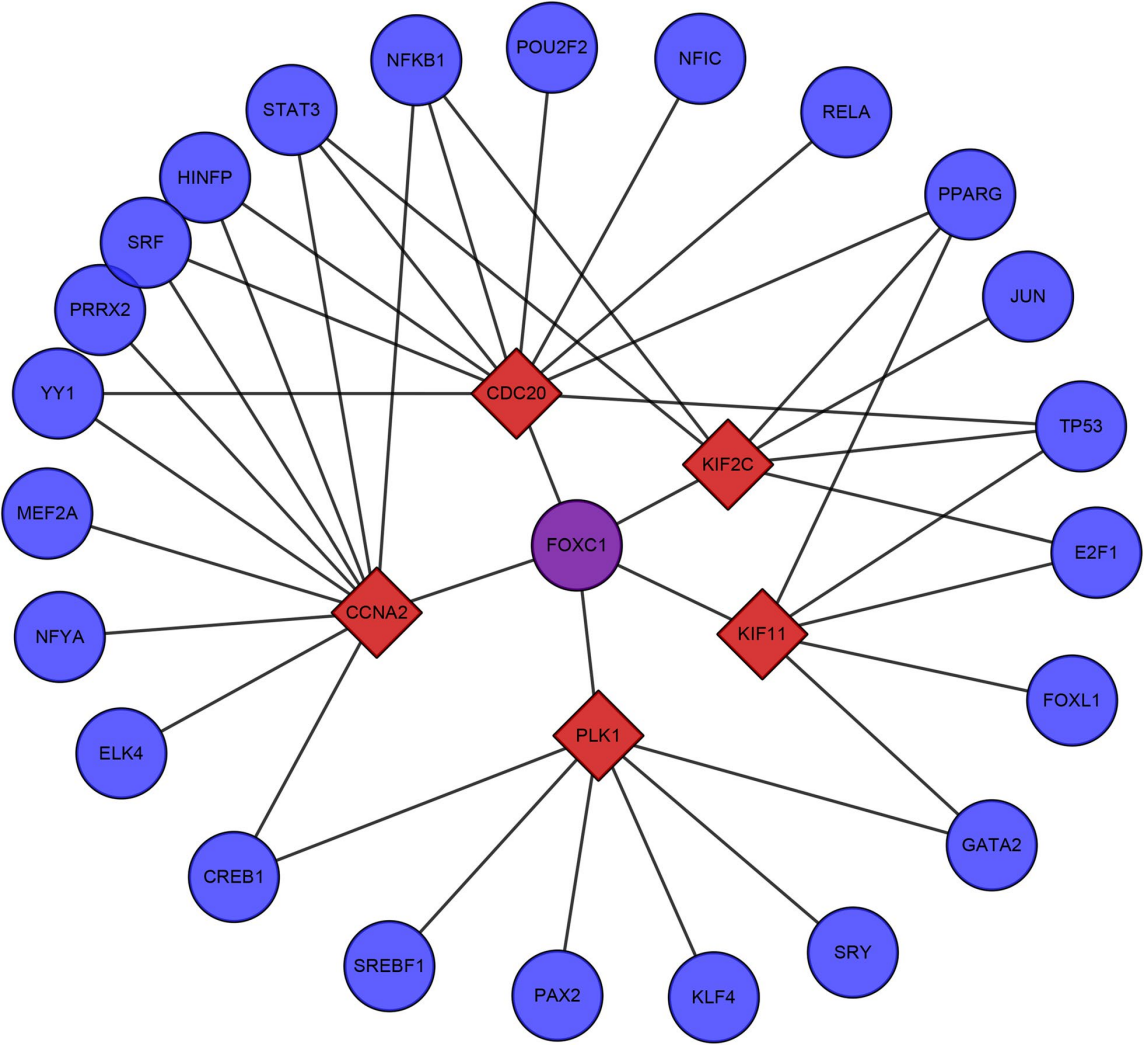
The hub gene-transcription factors (TF) regulatory network. Red diamond stands for the hub gene and circle node stands for the transcription factor**.** Purple node represents the key TF

Network Analyst website was used to construct TFs-genes intersection network and it is easier to identify the key TFs in the gene expression regulation. For the 5 identified hub genes, we established a TFs-genes regulatory network, which included 42 interaction pairs with 29 nodes (Fig. 5). From the TFs-genes interaction network graph, *CCNA2* and *CDC20* were both found to be influenced by 11 TFs, *PLK1* and *KIF2C* were affected by 7 TFs, and *KIF11* was regulated by 6 TFs according to the degree data. In addition, we also found that various TFs having the ability to regulate more than one hub gene, for example, STAT3 and NFKB1 both regulated 3 hub genes in the network. More important, based on TFs-genes network graph we found that *FOXC1* could regulate all of the hub genes, which meant *FOXC1* maybe an important transcription factor in the gene regulation in ECs responded to low shear stress treatment.

### Single-gene gene set enrichment analysis (GSEA)

**Fig. 6:**
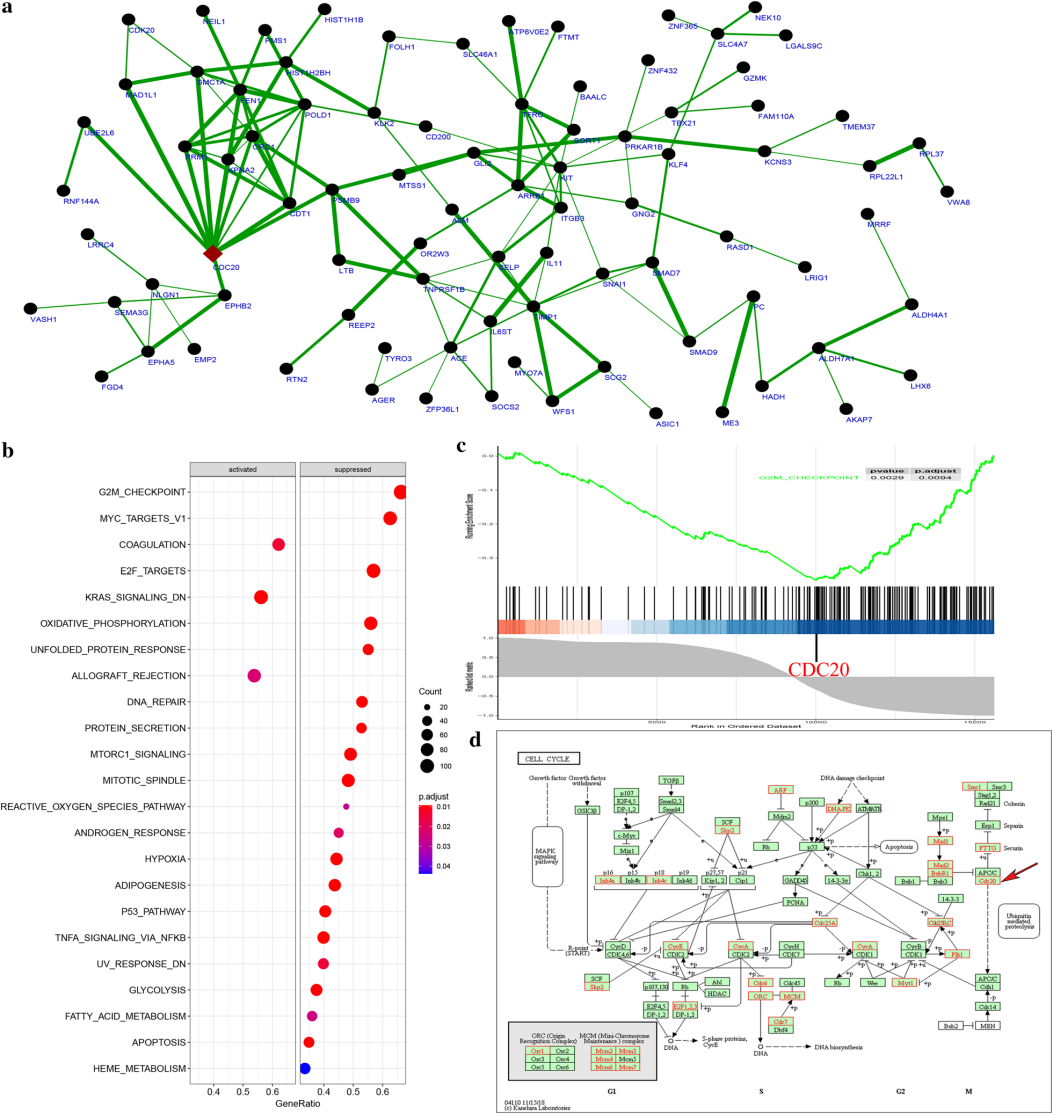
Single-gene GSEA analysis of *CDC20.* **a** The PPI network of genes with spearman correlation coefficient over 0.4 between *CDC20* and other genes in low shear stress treatment samples. Red diamond stands for *CDC20*. The thicker edge represents the higher correlation efficient. **b** GSEA pathway enrichment results of *CDC20* single-gene GSEA analysis. **c** Enrichment plot of G2M_CHECKPOINT pathway of *CDC20* single-gene GSEA analysis. **d** Pathway annotations of cell cycle pathway. Red label nodes represent down-regulated genes; green nodes have no significance. The author obtained copyright permission to use and modify the KEGG pathway map image hsa04110 Cell cycle—Homo sapiens (human)

The purpose of single-gene GSEA analysis was to find regulatory pathways or biological functions which were associated with the expression of interested genes. Considering that *CDC20* had the highest node degree in PPI of the DEGs and its relationship with key transcription factor *FOXC1*, we further performed pathway analysis of *CDC20* using single gene GSEA method. Briefly, the Spearman correlation coefficient between *CDC20* and other genes in low shear stress treatment samples was calculated and sorted by Spearman correlation coefficient. The PPI network of genes with spearman correlation coefficient over 0.4 between *CDC20* and other genes in low shear stress treatment samples was visualized by Cytoscape software. From the results (Fig. 6a), it displayed that *CDC20* has the highest connectivity degree compared with other genes. Further GSEA analysis results indicated that most of the enriched pathways were suppressed and G2M checkpoint pathway had the highest enrichment score with FDR < 0.25 and *P* value < 0.05 (Fig. 6b) and the top-scoring gene in the G2M checkpoint pathway was *CDC20* (Fig. 6c). We further investigated the gene expression in cell cycle pathway and found that most of the genes involved were down-regulated in this pathway of expression profile matrix. Two hub genes (*CDC20* and *PLK1*) were also found in this pathway. From the position of these two genes in cell cycle pathway, we could find that *CDC20* mainly participate in the ubiquitin mediated proteolysis process (Fig. 6d).

### The mRNA expression level of *CDC20* and *FOXC1* in ECs

**Fig. 7:**
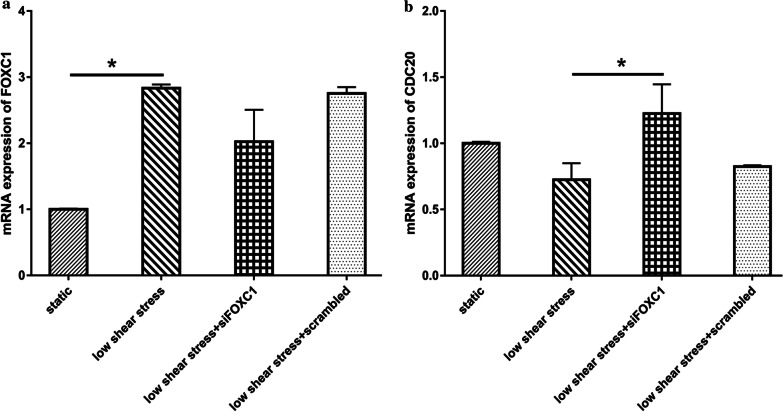
The mRNA expression level of *CDC20* and *FOXC1*. **a** The mRNA expression level of *FOXC1*. **b** The mRNA expression level of *CDC20*. Sample size (n = 3). Data were expressed as mean ± SD. * indicates *p* value < 0.05 compared to control

RT-PCR method was used to assess the mRNA expression profile of *CDC20* and *FOXC1*. As expected, the RT-PCR results demonstrated that the expression of *FOXC1* was obviously promoted in HUVECs exposed to low shear stress treatment which is consistent with the data of the microarray (Fig. 7a). In addition, from Fig. 7a we also certified that the expression level of *FOXC1* indeed decreased when transfected with siFOXC1.

From the bioinformatics analysis we speculated that *FOXC1* suppressed the expression of *CDC20*, in order to validate the hypothesis we compared the expression level of *CDC20* between HUVECs transfected with or without siFOXC1. First we compared the *CDC20* mRNA level between low shear stress and static groups, the results demonstrated that *CDC20* expression was inhibited by low shear stress treatment. This implied that there existed a negative correlation between *CDC20* and *FOXC1* and this trend was similar with that in the microarray data. Furthermore, the results indeed accorded with the hypothesis on the evidence that the mRNA expression level of *CDC20* was increased when HUVECs were transfected with siFOXC1 (Fig. 7b).

### Apoptosis of ECs treated with low shear stress

**Fig. 8:**
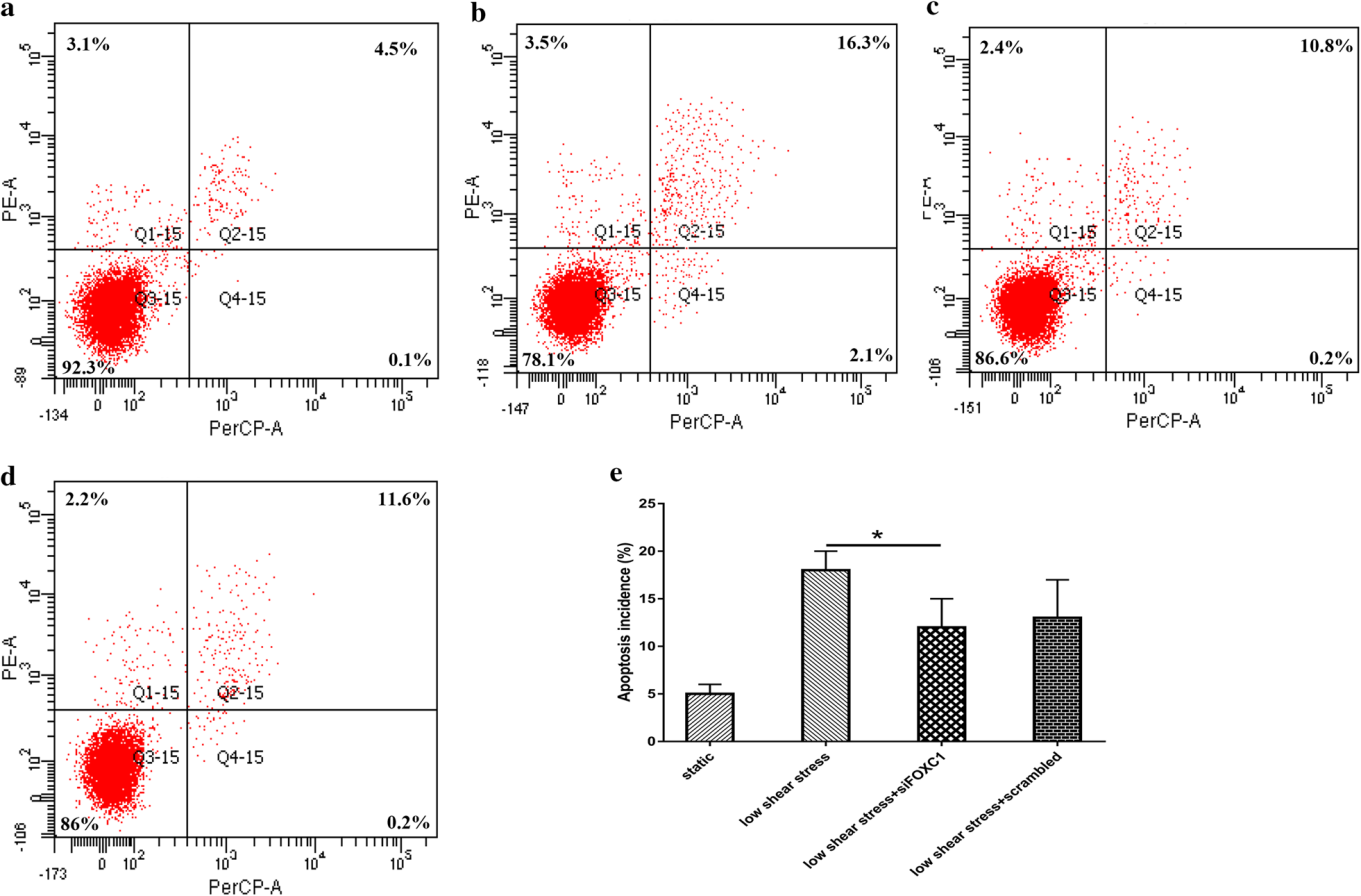
The apoptosis of HUVECs. The flow cytometry result of ECs treated with **a** static condition **b** low shear stress **c** low shear stress transfected with siFOXC1 **d** low shear stress transfected with siFOXC1 scrambled. **e** The bar graph of cytometry results. Sample size (n = 3). Data were expressed as mean ± SD. * indicates *p* value < 0.05 compared with control

Considering that these identified hub genes, especially *CDC20*, mainly participated in cell cycle, mitosis, chromosome separation pathway, we further evaluated the apoptosis incidence in ECs. As shown in Fig. 8a–e, we could find that low shear stress induced the apoptosis of HUVECs compared with static control group. More interestingly, when ECs were transfected with siFOXC1, the apoptosis incidence of ECs apparently decreased compared with the control group.

## Discussion

Based on the development of microarray and next generation sequencing technology, scientists could effectively identify various biomarkers and key molecular in various diseases. Amount of potential RNA and protein biomarkers have been predicted by bioinformatic analysis and validated by wet-lab experiment. The microarray dataset GSE16706 was generated by Conway [21] and he mainly compared the differential expressed genes between different shear stress treatments in HUVECs via parallel flow chamber device. However, the previous published paper did not investigate the regulatory network about these differential genes. Therefore, in my research, we re-analyzed the chip data and further demonstrated the regulatory mechanism of hub genes in ECs treated with shear stress treatment. Based on the new information of this paper, it will broaden our knowledge about cardiovascular disease. In this study, bioinformatic analysis was performed in HUVECs with low shear stress treatment compared with static treatment to find hub genes and potential TFs. We screened the expression matrix downloaded from GEO database and selected 652 DEGs from the datasets, including 333 up-regulated and 319 down-regulated genes. According to the topology parameters of PPI network, *CDC20*, *CCNA2*, *KIF11*, *KIF2C* and *PLK1* were selected as the hub genes. Furthermore, hub gene-TFs regulatory network revealed that *FOXC1* could regulate the entire hub genes indicating that *FOXC1* may be an important regulatory factor in HUVECs responded to shear stress treatment.

Conway [21] demonstrated that low average shear stress, including low shear stress and reversal flow, generated the similar gene expression changes compared with high shear stress. The author also indicated that shear stress mainly regulated the direction of gene changes in contrast with static culture, while the magnitude and waveform of shear stress determined the amplitude of gene expression change. Based on the previous knowledge and discovery of Conway, we also learned that low shear stress facilitated atherosclerosis and caused the most number of DEGs. Therefore, in this paper we mainly compared low shear stress with static condition to further investigate the gene regulation.

TFs regulate gene expression and play an essential role in almost all of the physiological process [22]. TFs are a group of protein molecules that could bind to the specific sequence of target genes and regulate gene expression at a time and space specific manner. By analyzing the characteristic of validated binding sites of TFs, we could predict the target gene by computational approaches [23]. Bioinformatic analysis method displayed important role in predicting new TFs and biomarkers in many diseases. In this paper, we identified related TFs with identified hub genes using NetworkAnalyst website which is thought to be a customer friendly and accurate tools to predict TFs. Our results demonstrated that these TFs screen out from NetworkAnalyst website formed a complicated regulatory network with identified hub genes. These results suggested that TFs may play multiple roles in regulating gene expression in ECs among cardiovascular disease.

*CDC20* is located in the 1p34.2 region of human chromosome, and the genome sequence contains 11 exons and 10 introns. *CDC20* is the activator of anaphase promoting complex/cyclosome (APC/C) which is an important constituent of the ubiquitin-proteolytic enzyme complex pathway [24]. *CDC20*, one of the earliest known cell cycle factors, whose main function is regulation of cyclin-B and the cell cycle regulator P21, and can affect the Wnt /β-catenin signaling pathway, Nek2A and the Kif18A pathway. *CDC20* also depletes endogenous PHF8 resulting in a prolonged G2 cycle and leads to mitotic defects [25]. *CDC20* plays an important role in guiding the ubiquitination and degradation of some proteins in the cell cycle and ensuring the normal separation of chromosomes [26]. During the cell division cycle, *CDC20* is the target of spindle assembly checkpoint and the positive regulator of the post-mitotic complex promotion. Previous study also indicated that shear stress influenced vascular endothelial cell proliferation by regulating cyclin-dependent kinase activity [27]. There also existed evidence suggested that exposure to laminar shear stress results in a reduction in endothelial cell rates of DNA synthesis and proliferation [28]. In this paper, we also identified that *CDC20* was the most significant hub gene and it was significantly down-regulated in the microarray expression matrix which is consistent with the previous study results. From the pathway analysis results and the annotation of cell cycle, we could find that *CDC20* mainly participated in the G2M checkpoint process and ubiquitin-mediated proteolysis process. Numerous cellular process regulated by ubiquitin-mediated proteolysis include the cell cycle, DNA repair and transcription and the immune response [29]. Defects in this proteolysis have a causal role in many human diseases, including a variety of cancer [30] and cardiovascular disease [31]. As well known, cell cycle disorders play an important role in the apoptosis in many cellular processes [32], this study results also displayed that the *CDC20* expression level had a similar variation trend with that of the apoptosis incidence in HUVECs treated with low shear stress. Based on this information, we implied that *CDC20* was an important biomarker in HUVECs responding to low shear stress.

We also search the references to investigate the function of other hub genes for the future research related with shear stress. *CCNA2* plays important role in S/G2 transformation and G2 phase checkpoint in human urinary bladder transitional cell carcinoma treated with fluid shear stress. It could induce cell cycle arrest and its function is consistent with the bioinformatics analysis in this paper. However, there is no relevant evidence that *CCNA2* could regulate cell cycle in endothelial cells [33]. Unfortunately, there was no relevant reference that supported the other three hub genes participated in the cell cycle regulation of shear stress treated endothelial cells. We also noticed that, among the five hub genes, *CCNA2* had the second highest connectivity. Therefore, these hub genes, especially *CDC20* and *CCNA2*, were worthy to be further investigate in the future.

*FOXC1* locates in chromosome 6p25.3 and it belongs to the forkhead family of transcription factors. Previous study revealed that *FOXC1* was involved in many biological processes, such as eye development [34], cancer [35], cardiovascular system development [36]. Previous studies certified that *FOXC1* mutations were associated with the defection of eye anterior segment [37] and cerebral small vessel disease [38]. Human patients with *FOXC1* mutations were associated with congenital heart disease [39]. In mice, knock-out of *FOXC1* in ECs impaired valve maturation [40]. There were experiments which demonstrated that shear stimulation of *FOXC1* regulated cytoskeletal activity [41]. In this study, we found that *FOXC1* had the highest connectivity with the entire hub genes which implied that *FOXC1* may have the ability to regulate multiple genes and play a pivotal role in low shear stress treated endothelial cells. In addition, we found that *FOXC1* was up-regulated in the expression profile matrix by log2FC equaled to 1.59, which implied that *FOXC1* may suppress the expression of *CDC20* in direct or indirect method. This hypothesis was supported by the results of the qRT-PCR of *CDC20* and *FOXC1* in wet experiment. However, there is still a lot of work to do to further validate the association of *FOXC1* and its target genes.

The proper selection of cell lines in cardiovascular disease research, especially in articles related to shear stress, is of great importance. The reasons for the choice of cell type (HUVEC) used in this paper are as follows. (a): HUVEC is a common used cell line in cardiovascular disease research, searching with key words ‘shear stress’ and ‘HUVEC’, there are hundreds of papers in PubMed. (b): The results derived from HUVECs were consistent with the results in animal experiment. HUVECs cell line was used in different types of shear stress and signal pathways in previous papers [42, 43]. However, I think the direct comparison of different cell types, including HUVECs and Arterial ECs, in shear stress research is very important in the future study.

The sample size of this paper was relatively small. Therefore, more microarray data and next generation sequencing data would be very important to help us study the regulatory mechanism in HUVECs subjected to shear stress treatment. In the paper we also identified other hub genes, but we did not further investigate these genes and its relationship with predicted TFs. This needs more work to do in the future.

## Conclusions

In conclusion, we identified five hub genes (*CDC20*, *CCNA2*, *KIF11*, *KIF2C* and *PLK1*) from the expression matrix downloaded from GEO datasets. Gene functional analysis suggested that biological functions of these hub genes mainly enriched in cell cycle. In addition, we identified a key transcription factor *FOXC1* which could regulate the entire hub genes. Single-gene GSEA analysis indicated that *CDC20* was linked to the G2M_CHECKPOINT pathway and cell cycle pathway. With the integrated bioinformatic analysis, a new transcriptional factor and hub-genes network related to endothelial cells treated with low shear stress were screened, and the new regulation mechanism we discovered may be potential therapeutic target for cardiovascular disease.

## Data Availability

GSE16706 dataset (including GSM418608, GSM418612, GSM418616, GSM418611, GSM418615 and GSM 418619) can be found in the Gene Expression Omnibus database (https://www.ncbi.nlm.nih.gov/geo/query/acc.cgi?acc=GSE16706).

## Abbreviations

TFs: Transcription factors
TFBSs: Transcription factor binding sites
HUVECs: Human umbilical vein endothelial cells
GEO: Gene Expression Omnibus
GO: Gene ontology
KEGG: Kyoto Encyclopedia of Genes and Genomes
PPI: Protein–protein interaction
DEGs: Differentially expressed genes
BP: Biological process
CC: Cellular component
MF: Molecular function
GSEA: Gene Set Enrichment Analysis
NCBI: National Center for Biotechnology Information
*CDC20*: Cell division cycle 20
*CCNA2*: Cyclin A2
*KIF11*: Kinesin family member 11
*KIF2C*: Kinesin family member 2C
*PLK1*: Polo like kinase 1
MCODE: Molecular Complex Detection
MSigDB: Molecular Signatures Database
APC/C: Activator of anaphase promoting complex/cyclosome
STRING: The search tool for the retrieval of interacting genes
DMEM: Dulbecco modified Eagle medium
FC: Fold change
